# Probing gait adaptations: The impact of aging on dynamic stability and reflex control mechanisms under varied weight-bearing conditions

**DOI:** 10.1007/s00421-025-05884-1

**Published:** 2025-07-01

**Authors:** Michelle Gwerder, Ursina Camenzind, Samira Wild, Yong K. Kim, William R. Taylor, Navrag B. Singh

**Affiliations:** 1 Laboratory for Movement Biomechanics, Department of Health Sciences and Technology, ETH Zürich, Gloriastrasse 37/39, 8092 Zurich, ZH Switzerland; 2 Singapore-ETH Centre, Future Health Technologies Program, CREATE Campus, Singapore, Singapore

**Keywords:** Aging, Dynamic stability, Gait adaptation, Gait variability, Reflex control mechanisms

## Abstract

**Purpose:**

Maintaining stable gait patterns is essential for preserving health and well-being throughout the aging process. While several biomechanical models have been developed to describe gait adaptation and stability, the role of reflex control mechanisms remains underexplored. This study aimed to understand the mechanisms by which disturbances to gait patterns (changes to weight-bearing conditions) are influencing gait adaptations, gait variability, and their underlying reflex control mechanisms during treadmill walking in young and older adults.

**Methods:**

Twenty young (mean age 25.7 ± 3.3 years) and 20 older adults (62.3 ± 4.3 years) walked on a treadmill under five weight-bearing conditions: normal bodyweight, 20 and 40% additional weight (bodyweight loading), and 20 and 40% reduced weight (bodyweight unloading). Linear mixed-effects models were used to assess spatiotemporal gait parameters, margin of stability, gait variability (standard deviation), and H-reflex amplitudes.

**Results:**

Bodyweight unloading significantly reduced antero-posterior margin of stability (*p* < 0.01). Compared to young adults, older adults exhibited shorter stride length, longer double-limb support time, larger antero-posterior margin of stability, and increased variability (*p* < 0.05). While H-reflex amplitudes increased with increasing weight-bearing in young adults, older adults were less capable to modulate their H-reflex amplitude across weight conditions.

**Conclusion:**

These findings suggest that gait adaptations under altered weight-bearing conditions involve shifts in temporal gait regulation and gait strategies. The reduced H-reflex modulation in older adults may indicate a limited ability to adapt spinal-level reflex amplitudes, leading to greater reliance on other balance control mechanisms. Understanding these neuromuscular adaptations is essential for designing prevention programs to enhance stability and prevent falls.

**Graphical abstract:**

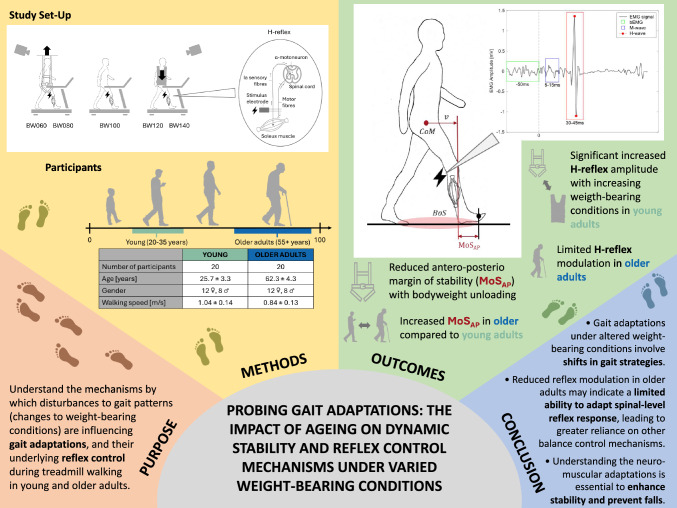

**Supplementary Information:**

The online version contains supplementary material available at 10.1007/s00421-025-05884-1.

## Introduction

Maintaining stable and functional gait patterns is essential for preserving health, autonomy, and well-being, particularly as we age (Studenski et al. 2011). However, age-related deterioration of physiological and sensorimotor systems results in gait adaptations and an increased risk of falls, which are among the leading causes of injury and loss of independence in older adults (Horak 2006; Iosa et al. 2014). Stable walking requires precise regulation of this dynamic process through the integration of complex sensorimotor control mechanisms (Al-Yahya et al. 2018; Clark 2015; Horak 2006; Yang and Gorassini 2006). These mechanisms work continuously to optimize our gait patterns, enabling the adaptation to various environmental challenges (Kulkarni et al. 2023). Control mechanisms can either operate anticipatorily, through a feedforward process, or retrospectively via sensory feedback. Research suggests that the most stable and robust walking is achieved by integrating feedforward and feedback control (Jin et al. 2023; Kuo 2002). The brain continuously processes proprioceptive, vestibular, and visual sensory input as feedback in order to adjust motor output accordingly (Reimann et al. 2019; Weerdesteyn et al. 2018). On the other hand, feedforward control relies on the brain’s ability to predict motor commands and execute them efficiently, ensuring smooth and consistent gait patterns (Weerdesteyn et al. 2018). Additionally, reflexes play a crucial role in gait control, enabling immediate and rapid real-time adjustments to maintain stability, adapt to changing conditions, and recover from disturbances (Capaday 2002; Zehr and Stein 1999). They play a vital role in fine-tuning gait patterns in response to both predicted and unexpected disturbances in the environment. This interplay of different control mechanisms is essential for achieving stable and efficient walking. Understanding the relationship between gait adaptations, underlying neural control mechanisms, and the influence of aging is critical for improving fall prevention aiming at maintaining mobility in older adults. Quantifying gait stability together with the underlying reflex control mechanisms provides valuable insights into control strategies employed towards foot placement to negotiate balance during walking and helps identify specific deficits in sensorimotor integration that arise with age.

One approach to evaluating walking stability involves modelling human locomotion as an inverted pendulum. Postural stability is maintained when the vertical projection of the centre of mass (CoM) remains within the base of support (BoS) defined by the feet in contact with the ground (Bruijn & van Dieen 2018; Winter 1995). However, this principle is violated during walking, as the CoM continuously moves beyond the BoS. To address this, Hof et al. (2005) introduced the concept of the extrapolated centre of mass (XCoM), which combines both the CoM position and the vertically projected proportion of its velocity. The margin of stability (MoS) is defined as the distance between the XCoM and the centre of pressure (CoP) (Curtze et al. 2024; Watson et al. 2021). In the absence of CoP data, the boundaries of the BoS can be approximated using foot marker positions (Watson et al. 2021). The MoS reflects how close a system is to losing balance (Hak et al. 2013). A positive MoS indicates that stability can be maintained within the current dynamic configuration, with higher values reflecting a greater margin for maintaining stability (Hof 2007). In contrast, a negative MoS implies that the XCoM lies outside the BoS, which may result in a fall if no correcting actions, such as a CoM adjustment or foot placement correction, are implemented (Hak et al. 2013; Hof 2007). Successfully detecting and correcting these abilities relies on a well-functioning control system that integrates both feedforward and feedback signals, allowing for rapid and precise postural adjustments.

Although the specific brain structures controlling gait remain unclear, research indicates that certain regions play a key role in modulating gait patterns, while visual feedback has little impact on gait variability regulation (Hausdorff 2005). Gait variability is an important aspect of walking control, representing a natural feature of human locomotion that is also linked to feedforward mechanism of balance control (Hausdorff 2005). Measures of gait variability serve as an important characteristic of gait adaptation, reflecting the redundancy and adaptability of human performance (Hausdorff 2005). Maintaining optimal variability within a certain range is essential for stability, allowing adaptation to environmental changes or task demands (König et al. 2016; Ravi et al. 2020). With aging, however, control systems decline, making balance regulation more challenging (Clark 2015). This decline often results in slower walking speed and increased gait variability, indicating diminished control and heightened risk of falls (McGibbon 2003). While gait variability reflects the adaptability and feedforward regulation of balance control, the role of reflexes in gait stability has not been previously examined but plausibly provides an insight into how subtle disturbances are regulated by the sensorimotor system. Gaining a deeper understanding of these reflexive mechanisms could therefore provide further insights into how stability is maintained, particularly in older adults, where sensory and motor control systems may be compromised.

The electrically evoked short-latency Hoffmann reflex (H-reflex) in the soleus muscle serves as a valuable tool for investigating neural adaptations in the human neuromuscular system (Chen and Zhou 2011; Zehr 2002). The H-reflex provides valuable insights into the functioning of spinal circuits and assesses the effectiveness of Ia afferents in evoking an action potential in motor neurons (Theodosiadou et al. 2023). Its amplitude is known to vary with postural demands, the phase of the gait cycle, and different weight-bearing conditions (Angulo-Kinzler et al. 1998; Capaday and Stein 1986; Cecen et al. 2018; Kim et al. 2024). As individuals age, H-reflex amplitude tends to decrease, reflecting a decline in spinal pathway excitability (Kido et al. 2004; Tsuruike et al. 2012). Additionally, while intra-task reflex modulation at different phases of walking is maintained (Chalmers and Knutzen 2002), reflex modulation across different tasks (lying vs. standing or different weight-bearing conditions during standing) becomes less pronounced in older compared to young adults (Angulo-Kinzler et al. 1998; Koceja & Mynark 2000; Scalia et al. 2024; Tsuruike et al. 2012). The age-related decline in reflex amplitude indicates a reduced capacity of the nervous system to rapidly and effectively adjust to postural challenges. However, our understanding of how reflex control mechanisms integrate biomechanically to regulate gait stability while walking remains limited. Gaining deeper insights into the neural reflex mechanisms that regulate walking and how they evolve with age is crucial for improving gait stability in older adults. Such knowledge can support more effective strategies to mitigate fall risk, ultimately enhancing quality of life and maintaining autonomy in this population.

The aim of this study was therefore to examine how varying weight-bearing conditions influence gait adaptations, gait variability, and their underlying H-reflex mechanisms during treadmill walking in young and older adults. We hypothesized that H-reflex and gait variability might indicate adaptation potential in young and older participants.

## Materials and methods

### Participants

This study was approved by the Zurich cantonal ethics committee (BASEC-No 2019-00678) and performed in accordance with the Declaration of Helsinki. All participants provided written informed consent prior to the measurement.

Twenty healthy young (between 20 and 35 years) as well as 20 healthy older adults (55 years and older) participated in this study (Table 1). Inclusion criteria were (a) no acute or chronic neurologic or musculoskeletal diseases (e.g. epilepsy, stroke, Multiple Sclerosis or Parkinson’s disease), (b) no injuries or surgeries to the lower extremities in the last five years, (c) no vertigo or balance problems, (d) no use of medications affecting balance, and (e) no alcohol 48 h, and no strenuous exercise within 24 h prior to the measurement.

**Table 1:** Anthropometrics of young and older participants (mean ± SD)

	Young adults	Older adults
Number	20	20
Gender	8 M, 12 F	8 M, 12 F
Age [years]	*25.7 ± 3.3*	*62.3 ± 4.3*
Height [cm]	173.2 ± 8.9	170.9 ± 9.2
Weight [kg]	67.7 ± 9.8	67.2 ± 10.3
Walking speed [m/s]	*1.04 ± 0.14*	*0.84 ± 0.13*

Italic denote significant group differences between young and older adults

### Experimental setup and procedure

Participant demographics, including age, height, and weight were recorded before identifying the optimal location to evoke an H-reflex. To standardize the measurement, electrodes were placed and confirmed in a prone position (see sect. “Locating the H-reflex”). Each participant’s specific stimulus intensity was then determined using H-reflex recruitment curves (RC, sect. “Recruitment curve”) while walking on a treadmill (h/p/cosmos quasar, h/p/cosmos sports & medical GmbH, Germany). Once the stimulus intensity was established, participants were assessed under various weight-bearing conditions.

#### H-reflex

##### Locating the H-reflex

The H-reflex was electrically elicited in the dominant leg, determined by first asking participants:”If you were kicking a ball, which leg would you use to shoot the ball?” (van Melick et al. 2017). A wireless electromyography (EMG) electrode (PicoEMG, Cometa, Italy, 3000Hz sampling frequency) was then positioned on the soleus muscle of the dominant leg following the “surface electromyography for the non-invasive assessment of muscles” (SENIAM) guidelines (Hermens et al. 2000). The anode (Disposable Adhesive Surface Electrode, Spes Medica, Italy) was placed on the front of the leg, two centimetres above the patella. With 20° knee flexion, a line was drawn across the popliteal fossa. The tibial nerve, typically located approximately 1.5cm lateral to the midpoint of this line (Ozyurt et al. 2018), was then identified using a movable electrode, with a rectangular pulse (1ms duration) delivered by a constant current stimulator (DS7AH, Digitimer, UK). Once the H-reflex was successfully elicited (visible H-wave in EMG signal), a self-adhesive stimulation cathode (Ag/AgCl Kendall™ H124SG, CardinalHealth, Switzerland) was securely fixed at the location identified by the movable electrode. The H-reflex amplitude was then verified in the EMG signal with the participant in a standing position.

##### Timing of H-reflex stimuli

During walking, stimuli were delivered between heel strike and mid-stance, the phase previously identified as the most sensitive for H-reflex modulation (Chalmers and Knutzen 2000; Krauss and Misiaszek 2007). Gait events were detected in real-time using adapted Matlab scripts (Matlab 2018a, The Math Works, USA), based on kinematic data from foot markers of the dominant leg (see sect. “Data analysis”) (O’Connor et al. 2007). To prevent predictability, motor neuron saturation or under-recruitment, as well as to avoid post-activation depression and habituation to stimulus timing, stimuli were applied at randomized intervals ranging from 9 to 16s between pulses (Chen and Zhou 2011; Pierrot-Deseiligny and Mazevet 2000).

##### Recruitment curve

**Fig. 1:**
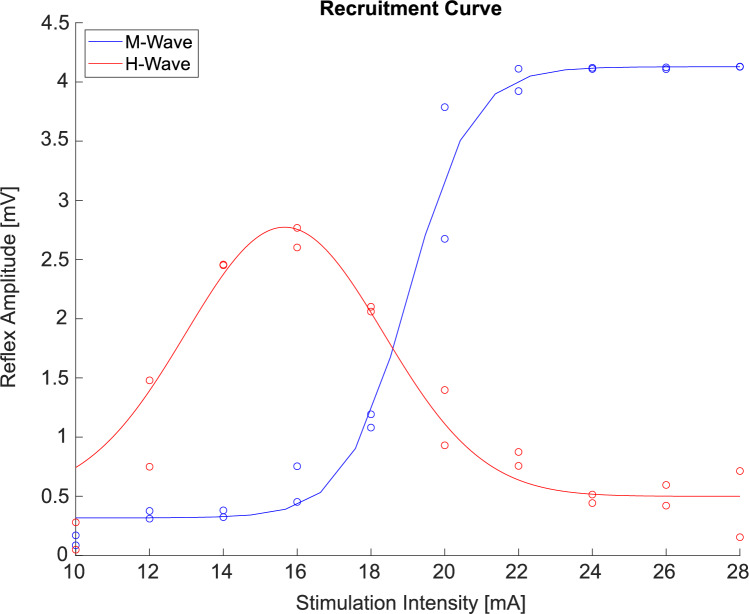
H-reflex recruitment curve (red) and corresponding M-wave (blue) of one young, representative participant. With increasing stimulation intensity, the H-reflex amplitude starts to increase. Later, the M-wave starts to increase with a corresponding decrease in H-reflex

To determine the H-reflex recruitment curve (RC) during walking (Fig. 1), stimulation began at 10-15mA and was increased in subject-specific increments of 0.5-2mA every second stimulus until the M-wave peak-to-peak amplitude reached a plateau (*M*_max_). Peak-to-peak amplitudes of the H-reflexes and M-waves were calculated, and gaussian and sigmoid functions were fitted to the data. Stimulus intensity was then standardized to 80% of each participant’s individual maximum H-reflex amplitude (*H*_max_). This intensity was selected because it is known to reliably produce a sufficiently large and reproducible reflex response that is sensitive to changes, while also avoiding stimulation during the descending phase of the H-reflex curve to mitigate the risk of antidromic collision of the two pulses (Bouguetoch et al. 2020; Palmieri et al. 2004; Scalia et al. 2024; Theodosiadou et al. 2023).

#### Gait analysis

**Fig. 2:**
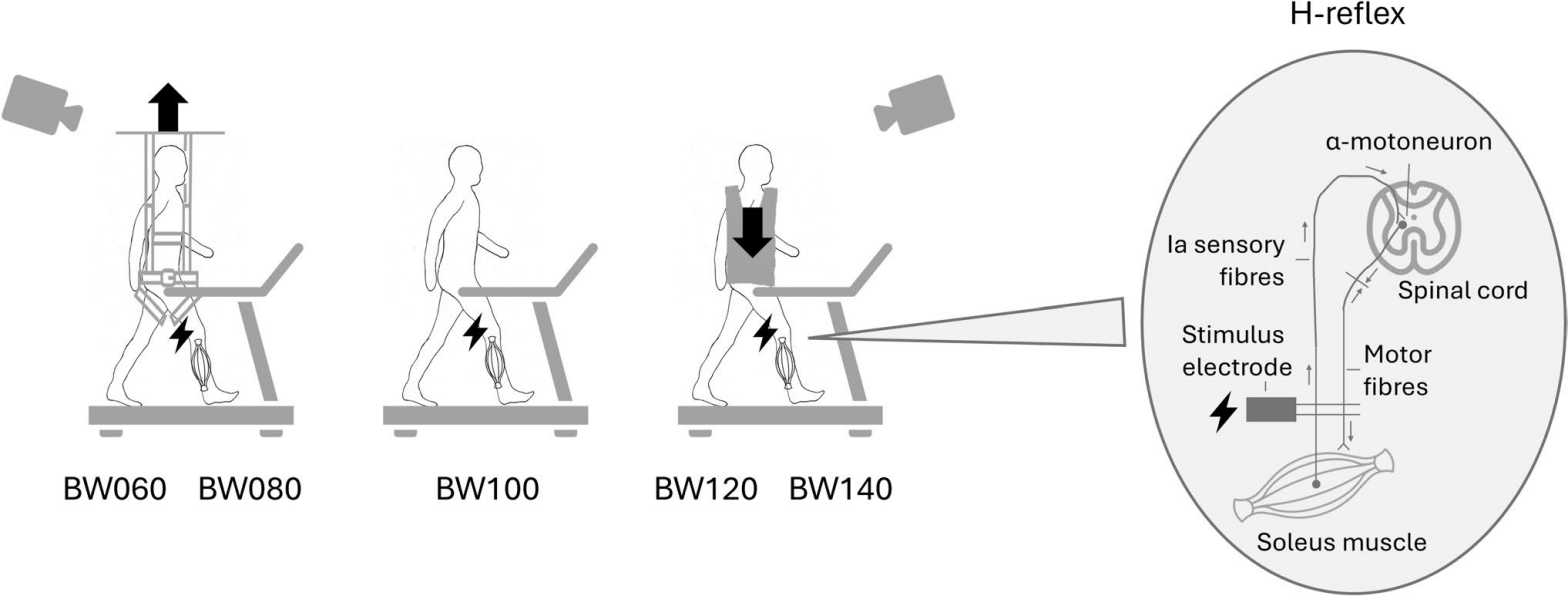
Study setup: Participants walked barefoot on a treadmill under five different weight-bearing conditions: normal bodyweight (BW100), 40% bodyweight unloading (BW060), and 20% bodyweight unloading (BW080), achieved using a bodyweight support system, as well as 20% additional bodyweight (BW120), and 40% additional bodyweight (BW140), applied using a weight vest. On the right, the H-reflex mechanism is illustrated, showing the stimulus electrode triggering the nerve in the back of the knee, the Ia sensory fibres connecting with the α-motoneuron in the spinal cord, projecting to the soleus muscle fibres

Participants were equipped with 62 reflective markers, sampled at a frequency of 200Hz using a 10-camera optical motion-capture system (VICON Motion Systems Ltd, Oxford, UK) (Kim et al. 2024) (Supplementary Material, 1. Marker Set). To investigate the effect of weight-bearing on gait adaptations and H-reflex control mechanisms, subjects walked barefoot on the treadmill while wearing a safety harness. At the beginning of each measurement, participants familiarized themselves with the treadmill and selected a comfortable walking speed, which was maintained across all conditions. Five weight-bearing conditions were tested in a randomized order, with each lasting for six minutes (Fig. 2): (1) baseline condition with normal bodyweight (BW100), (2) 20% additional bodyweight (BW120) applied using a weight vest (EZ Vest Pro, Kensui LLC, US), (3) 40% additional bodyweight (BW140), (4) 20% bodyweight unloading (BW080) achieved using a bodyweight support system (Ergolet Walking Sling, Winncare Nordic ApS, DK), and (5) 40% bodyweight unloading (BW060). Marker kinematic data and EMG signals were measured to determine gait events, spatiotemporal gait parameters, MoS, gait variability, and H-reflex amplitudes.

### Data analysis

To ensure analysis of steady-state walking, the first and last 30 s of each six-minute walking trial were excluded. Kinematic marker data were low-pass filtered using a Butterworth filter (zero-phase lag, fourth-order, 25Hz cut-off frequency) before gait events (heel-strike and toe-off) were identified using a foot velocity algorithm (O’Connor et al. 2007). Foot marker trajectories were used to calculate the vertical velocity of the foot, which enabled the identification of heel strike and toe-off events. A stride was defined as two consecutive heel strikes of the same leg. In addition to step width and double-limb support time, the other spatiotemporal gait parameters calculated for each leg and stride independently included: stride time, single-limb support time, and stride length. The mean and standard deviation (SD) were calculated, with SD serving as a measure of variability to assess stride-to-stride consistency. As no significant differences were observed between parameters from the two legs, spatiotemporal gait parameters were only analysed for the stimulated leg.

#### Dynamic stability

To assess dynamic stability, the inverted pendulum model of Hof et al. (2005) was used. The body’s instantaneous whole-body centre of mass ($\text{CoM}$) was approximated from the centroid of seven pelvis marker positions, while the extrapolated centre of mass ($\text{XCoM}$) was calculated as follows:

$$\text{XCoM}=\text{CoM}+ \frac{{\text{CoM}}_{\text{v}}}{\sqrt{\frac{g}{l}} }$$

where ${\text{CoM}}_{\text{v}}$ is the velocity of the $\text{CoM}$ adjusted for treadmill walking, $g$ is the gravitational constant $g=9.81 \frac{\text{m}}{{\text{s}}^{2}}$ and $l$ is the length defined as the distance from the $\text{CoM}$ to the midpoint between the two malleoli markers of the ankle. For instantaneous mechanical stability, $\text{XCoM}$ should be located within the base of support ($\text{BoS}$), hence providing a margin of stability ($\text{MoS}$):

$$\text{MoS}=\text{BoS}-\text{XCoM}$$

$\text{MoS}$ was calculated at heel strike in the antero-posterior (${\text{MoS}}_{\text{AP}}$) and medio-lateral directions (${\text{MoS}}_{\text{ML}}$), selected as the instant that determines foot placement for the stance phase (Hof 2008; Sangeux et al. 2024; Watson et al. 2021) and shortly before eliciting an H-reflex. Boundaries of the $\text{BoS}$ were defined by the first metatarsal marker for antero-posterior and the fifth metatarsal marker for the medio-lateral directions, respectively.

#### H-reflex

**Fig. 3:**
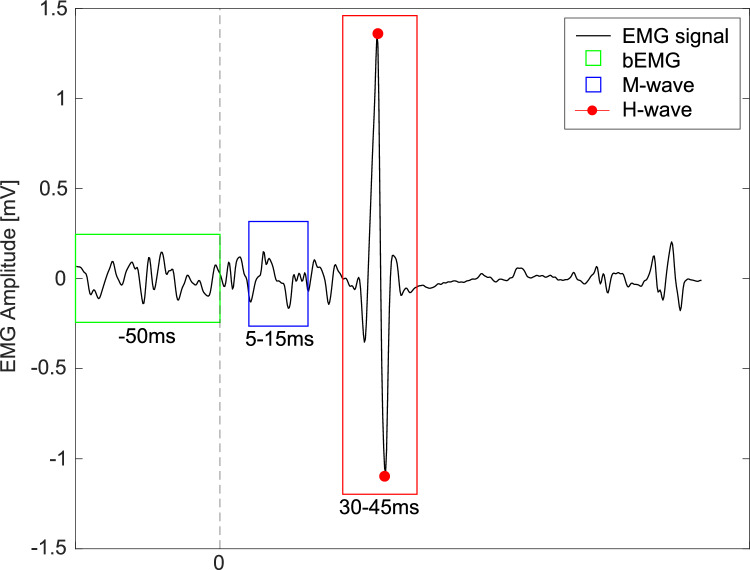
Example of a soleus EMG signal (black) from a young participant. The dashed line at 0 marks the timing of the electrical stimulus. The green box indicates the window used to calculate average background EMG (bEMG), the blue box represents the time window for the M-wave, and the red box shows the peak-to-peak amplitude of the H-reflex

Peak-to-peak amplitudes of the H-reflex (30–45ms after stimulus) of the raw, non-rectified EMG signal were calculated for each of on average 23 stimuli per participant and condition (exemplarily shown in Fig. 3). A few outlier stimulations occurred outside the early stance phase mainly due to marker occlusions interfering with trigger timing, which were excluded from the analysis (Simonsen and Dyhre-Poulsen 1999). Due to limitations in the recording system, the maximum stimulation intensity in some participants in the RC did not reach a true M_max_, but rather the system’s maximum recording capacity. As a result, normalization to M_max_ was not feasible for all participants. To maintain consistency and ensure data comparability, the inclusion of H-reflex amplitudes was dependent upon stable muscle activity during the M-wave time window. Specifically, the EMG amplitude within this window was required to fall within ± 20% of the condition-specific mean (Kim et al. 2024). Furthermore, H-reflex amplitudes were normalized to the average background EMG (bEMG) activity recorded during the 50ms preceding the stimulation but following heel strike (H-reflex_norm_). This approach has been employed in comparable studies to reflect reflex excitability relative to ongoing muscle activation (Kim et al. 2024; Palmieri et al. 2004).

### Statistical analysis

Linear mixed-effects models were used to analyse spatiotemporal gait parameters, MoS, gait variability, and H-reflex adaptations to different weight-bearing conditions and age. The dependent measures included mean and variability (SD) parameters of stride time, single- and double-limb support time, stride length, step width, MoS_AP_ and MoS_ML_, as well as H-reflex, H-reflex_norm_, and bEMG. Age group (young and older adults) and bodyweight condition (BW060, BW080, BW100, BW120, BW140) were treated as fixed effects, along with their interaction, while participants were treated as random effects, with baseline set at BW100 for comparisons. Significance was set at *p* ≤ 0.05, but was adjusted for multiple comparisons where appropriate using the false discovery rate method (Benjamini and Hochberg 1995). All analyses were performed using RStudio (v2024.04.2, R Core Team, Austria).

## Results

### Mean gait adaptations

On average, 258 strides per participant and condition were analysed. Single- and double-limb support time exhibited significant differences for bodyweight unloading conditions (BW060, BW080) compared to the baseline condition (BW100) for both groups (*p* < 0.01; Table 2 and Supplementary Material S1). In the BW060 condition, single-limb support time increased by 17% in young and 14% in older participants, while double-limb support time decreased by 47% and 31% respectively. Stride length in the young group was 3% shorter in BW060 (*p* < 0.01), and step width in the BW080 condition was reduced by 11% in young and 15% in older participants (*p* < 0.05). Post-hoc comparisons revealed a significant reduction in MoS_AP_ in both bodyweight unloading conditions for both groups (*p* < 0.001) compared to BW100, with the greatest decrease of 58% observed in young participants under the BW060 condition.

**Table 2:** Spatiotemporal gait parameters and margin of stability (mean ± SD) for the five different weight-bearing conditions in young and older participants

	Young adults				
	BW060	BW080	BW100	BW120	BW140
Stride time [s]	1.12 ± 0.10	1.13 ± 0.09	1.13 ± 0.08	1.13 ± 0.08	1.12 ± 0.08
Single-limb support [s]	0.48 ± 0.05***	0.45 ± 0.03***	0.41 ± 0.03	0.41 ± 0.02	0.40 ± 0.02**
Double-limb support [s]	0.16 ± 0.05***	0.23 ± 0.04***	*0.30 ± 0.04*	*0.31 ± 0.04*	0.33 ± 0.04**
Stride length [cm]	115.8 ± 13.3**	*117.3 ± 14.9*	*118.8 ± 14.8*	*119.1 ± 14.2*	*118.6 ± 13.8*
Step width [cm]	8.8 ± 2.0	8.4 ± 2.3*	9.4 ± 2.3	9.0 ± 2.1	9.3 ± 2.4
MoS_AP_ [cm]	6.5 ± 3.2***	10.9 ± 3.3***	*15.6 ± 2.9*	*16.1 ± 2.9*	*16.0 ± 2.9*
MoS_ML_ [cm]	9.2 ± 2.1	9.1 ± 2.0	9.8 ± 2.1	9.9 ± 2.0	10.0 ± 2.1

	Older adults				
	BW060	BW080	BW100	BW120	BW140
Stride time [s]	1.22 ± 0.20	1.21 ± 0.18	1.21 ± 0.15	1.20 ± 0.16	1.19 ± 0.17
Single-limb support [s]	0.49 ± 0.07***	0.46 ± 0.06***	0.43 ± 0.05	0.42 ± 0.05	0.41 ± 0.06***
Double-limb support [s]	0.24 ± 0.08***	0.29 ± 0.08***	*0.35 ± 0.07*	*0.37 ± 0.07*	0.38 ± 0.06**
Stride length [cm]	*100.6 ± 12.9*	*100.4 ± 11.2*	*101.8 ± 11.2*	*101.7 ± 11.1*	*101.5 ± 12.0*
Step width [cm]	9.2 ± 3.2	8.2 ± 3.2***	9.6 ± 3.3	9.1 ± 3.1	9.2 ± 3.7
MoS_AP_ [cm]	12.6 ± 3.5***	15.1 ± 3.5***	*19.1 ± 2.9*	*19.7 ± 3.3*	*19.6 ± 2.7*
MoS_ML_ [cm]	9.3 ± 2.4	9.0 ± 2.6	9.8 ± 1.9	9.7 ± 1.9	9.9 ± 1.8

*MoS*_*AP*_ antero-posterior margin of stability, *MoS*_*ML*_ medio-lateral margin of stability

Pairwise post-hoc comparison tests highlight significant differences from baseline (BW100) (*p < 0.05, **p < 0.01, ***p < 0.001) and italic denote significant group differences between young and older adults

Single-limb support time decreased by 2% in young and 5% in older participants for the highest bodyweight loading condition (BW140), while double-limb support time increased by 10% and 9%, respectively.

Across all conditions, group-level differences were observed in three parameters: older adults showed significantly longer double-limb support time (*p* < 0.05), increased MoS_AP_ (*p* < 0.01), and shorter stride length (*p* < 0.001) compared to young adults (Table 2 and Supplementary Material 2. Statistical Analysis, table S1).

### Gait variability

In the bodyweight unloading condition BW060, variability in single- and double-limb support times, and MoS_ML_ increased compared to baseline (*p* < 0.001; Table 3 and Supplementary Material S2). Step width variability was significantly reduced in both BW060 and BW080 conditions by 32% and 23% in young and by 25% and 30% in older participants, respectively.

**Table 3:** Gait variability (mean ± SD) for the five different weight-bearing conditions in young and older participants

	Young adults				
	BW060	BW080	BW100	BW120	BW140
Stride time [ms]	21.0 ± 6.9*	*16.4 ± 6.1*	*15.9 ± 7.8*	*16.8 ± 7.0*	*16.1 ± 5.4*
Single-limb support [ms]	15.8 ± 8.0***	*10.4 ± 3.2*	*8.8 ± 4.6*	*8.7 ± 4.0*	*8.4 ± 3.6*
Doubl-limbe support [ms]	17.1 ± 6.6***	*12.2 ± 3.0*	*11.2 ± 3.8*	*11.2 ± 3.5*	*12.2 ± 4.1*
Stride length [cm]	*3.1 ± 0.8*	*2.6 ± 0.7*	*2.8 ± 0.8*	*2.9 ± 0.9*	*2.9 ± 0.7*
Step width [cm]	1.3 ± 0.6**	1.5 ± 0.5*	1.9 ± 0.7	2.1 ± 0.6	2.1 ± 0.8
MoS_AP_ [cm]	1.4 ± 0.4	1.2 ± 0.3	1.3 ± 0.4	1.4 ± 0.4	1.5 ± 0.4
MoS_ML_ [cm]	1.2 ± 0.4***	1.0 ± 0.2	0.9 ± 0.3	0.9 ± 0.2	1.0 ± 0.3

	Older adults				
	BW060	BW080	BW100	BW120	BW140
Stride time [ms]	*30.0 ± 13.2*	*25.9 ± 12.8*	*28.9 ± 16.9*	*28.3 ± 19.1*	*28.3 ± 13.3*
Single-limb support [ms]	20.3 ± 10.0***	*14.5 ± 5.8*	*14.8 ± 7.2*	*15.1 ± 9.0*	*15.1 ± 7.2*
Double-limb support [ms]	22.0 ± 9.5**	*18.0 ± 9.2*	*18.4 ± 9.1*	*17.9 ± 8.8*	*18.2 ± 8.7*
Stride length [cm]	*3.6 ± 0.9*	3.1 ± 0.8*	*3.6 ± 1.0*	*3.5 ± 0.9*	*3.5 ± 0.85*
Step width [cm]	1.6 ± 0.8**	1.5 ± 0.5***	2.2 ± 0.7	2.5 ± 0.9	2.7 ± 0.9
MoS_AP_ [cm]	1.8 ± 0.6	1.6 ± 0.5	1.7 ± 0.5	1.7 ± 0.4	1.7 ± 0.4
MoS_ML_ [cm]	1.3 ± 0.5**	1.2 ± 0.4	1.1 ± 0.4	1.1 ± 0.4	1.1 ± 0.3

*MoSAP* antero-posterior margin of stability, *MoSML* medio-lateral margin of stability

Pairwise post-hoc comparison tests highlight significant differences from baseline (BW100) (*p < 0.05, **p < 0.01, ***p < 0.001) and italic denote significant group differences between young and older adults

For bodyweight loading conditions, no significant differences in variability were observed compared to baseline.

Older participants exhibited significantly higher variability in four spatiotemporal gait parameters (stride time, single- and double-limb support times, and stride length; all *p* < 0.05) in comparison to their younger counterparts. The largest group difference was observed in stride length variability (Table 3 and Supplementary Material 2. Statistical Analysis, table S2).

### H-reflex

For the total 4651 elicited reflexes, 3313 (71%) were included in the analysis and the included stimuli were applied on average at 19.3% of the gait cycle or 0.22s ± 0.08s after heel strike. In the young group, 1762 out of 2358 recorded reflexes (75%) were included in the final analysis, while in the older adult group, 1551 out of 2293 reflexes (68%) were retained.

Post-hoc comparisons in bEMG amplitude revealed significant differences from baseline for all bodyweight conditions except BW120 (*p* < 0.01) in young participants, while no significant differences were observed in older adults across all conditions (Table 4 and Supplementary Material 2. Statistical Analysis, table S3). Furthermore, no significant differences for bEMG were observed between the two groups.

**Table 4:** H-reflex, normalized H-reflex (H-reflex_norm_), and baseline EMG (bEMG) activity (mean ± SD) under the five different weight-bearing conditions in young and older adults

	Young adults				
	BW060	BW080	BW100	BW120	BW140
H-reflex [mV]	0.62 ± 0.37***	0.72 ± 0.44***	1.08 ± 0.54	1.25 ± 0.74	1.35 ± 0.77*
H-reflex_norm_	35.08 ± 16.23	37.20 ± 16.24	38.85 ± 20.88	38.21 ± 23.83	33.14 ± 20.87
bEMG [mV]	0.02 ± 0.02**	0.03 ± 0.02*	0.05 ± 0.06	0.05 ± 0.06	0.06 ± 0.07*

	Older adults				
	BW060	BW080	BW100	BW120	BW140
H-reflex [mV]	0.64 ± 0.30	0.69 ± 0.40	0.74 ± 0.45	0.82 ± 0.50	0.89 ± 0.52
H-reflex_norm_	31.79 ± 21.79	41.48 ± 32.62	32.53 ± 16.90	31.11 ± 16.45	28.05 ± 15.30
bEMG [mV]	0.03 ± 0.02	0.02 ± 0.01	0.03 ± 0.01	0.03 ± 0.02	0.04 ± 0.03

Pairwise post-hoc comparison tests highlight significant differences from baseline (BW100) (*p < 0.05, **p < 0.01, ***p < 0.001)

For H-reflex in young participants, BW060, BW080, and BW140 all showed significant differences compared to baseline (*p* < 0.05; Table 4 and Supplementary Material S3). H-reflex amplitudes decreased by 43% and 32% under bodyweight unloading conditions (BW060 and BW080) and increased by 22% under bodyweight loading (BW140). In contrast, older adults exhibited no reflex modulation across the different bodyweight conditions (Table 4). An interaction effect was observed between the older age group and the BW060 condition compared to baseline (Supplementary Material S3).

H-reflex_norm_ (adjusted for bEMG) showed no significant differences between groups or weight conditions.

## Discussion

A deeper understanding of how reflex control mechanisms contribute to gait stability is crucial for developing fall prevention strategies, improving quality of life, and maintaining autonomy in older adults. This study examined how healthy young and older participants adapt to different weight-bearing conditions, focusing on dynamic stability and underlying H-reflex control mechanisms during treadmill walking. Our findings indicate that bodyweight unloading led to longer single-limb support time, shorter double-limb support time, and decreased MoS_AP_, suggesting adaptations in temporal gait regulation and a shift in gait strategy to accommodate varying reduced weight-bearing conditions. Across all conditions, older adults demonstrated lower stride length, prolonged double-limb support time, higher MoS_AP_, and greater gait variability compared to younger adults, reflecting a preference towards conservative gait as well as potential impairments in motor control. The increased H-reflex amplitudes observed with higher weight conditions in young adults suggest physiologically responsive reflex modulation to varying loading demands. In contrast, the absence of reflex modulation in older adults may indicate a reduced ability to modulate spinal-level reflex amplitudes to changing weight-bearing conditions, increasing the need for compensation through other mechanisms to regulate balance control (Weaver et al. 2012).

### Mean gait adaptations

In this study, under bodyweight unloading conditions, participants had a significant increase in single-limb support time and a corresponding decrease in double-limb support time compared to baseline, suggesting adaptations in temporal gait regulation to accommodate reduced bodyweight (Herssens et al. 2021). During single-limb support, the CoM follows an inverted pendulum trajectory. A prolonged single-limb support phase compared to baseline suggests that CoM progression and corresponding foot placement may occur at a slower pace (Adamczyk and Kuo 2009). Conversely, the reduced double-limb support time limits the duration available for CoM redirection to the next step, thereby restricting opportunities for corrective adjustments (Adamczyk and Kuo 2009; Williams and Martin 2019). This may result in less efficient and more variable gait, as the redirection of the CoM occurs over a shorter period, potentially increasing the peak force and power demands on the muscles (Adamczyk and Kuo 2009). Additionally, the MoS_AP_ decreased under bodyweight unloading conditions in comparison to baseline, suggesting an adapted gait strategy. A smaller MoS_AP_ reflects reduced forward momentum, which may indicate diminished forward propulsion and weaker push-off forces from the stance leg. Such lower push-off forces could impair the ability to project the CoM forward effectively, making it more challenging to maintain stability in the antero-posterior direction (Kulkarni et al. 2023). For medio-lateral direction, this study found a significantly smaller step width in the BW080 condition compared to baseline, suggesting that moderate bodyweight unloading reduces the BoS in the frontal plane. However, greater unloading may introduce an unfamiliar and more challenging condition, leading to a compensatory increase in BoS through adapted foot placement to enhance stability. Through this adaptation, participants may enhance their balance at the expense of increased energy expenditure (Donelan et al. 2002). Given the strong correlation between step width and MoS_ML_ (Herssens et al. 2020), it is unsurprising that MoS_ML_ exhibited similar trends, even though these changes were not statistically significant. MoS_ML_ is influenced by multiple factors, and participants may have employed alternative compensatory strategies to maintain stability. Rather than relying solely on step width adjustments, they may have adapted through e.g. changes in trunk control or hip strategy (Afschrift et al. 2018; Horak and Nashner 1986). These compensatory mechanisms may have mitigated any expected reductions in MoS_ML_, hence explaining the absence of a significant decrease.

Under bodyweight loading conditions, participants appeared to minimize single-limb support time, transitioning quickly into the more stable double-limb support phase through precise push-off timing (Bauby and Kuo 2000). This strategy aligns with adjustments to push-off mechanics for correcting foot placement errors during steady-state walking, where both accurate timing and appropriate force application are critical for maintaining stability (Jin et al. 2023). Additionally, spending less time on one leg reduces the duration available for the CoM to follow an unplanned or unstable trajectory. The resulting shorter swing phase increases CoM velocity in the antero-posterior direction, as the same pendulum distance (with stride length and walking speed remaining unchanged from baseline) must be covered in a shorter time. This leads to greater energy demand. The higher forces required for redirecting the CoM at foot contact contribute to increased step-to-step energy costs and greater push-off force requirements, hence lowering gait stability control (Donelan et al. 2002; Kuo and Donelan 2010). The increased energy demand and higher forces required for CoM redirection may lead to greater muscular fatigue over prolonged walking, potentially reducing efficiency and increasing fall risk. Additionally, reduced stability control could make individuals more susceptible to balance disturbances, requiring greater neuromuscular effort to recover from perturbations, which may not always be successfully executed in populations with impaired motor control.

Older adults exhibited significantly longer double-limb support time, shorter stride length, and higher MoS_AP_ compared to younger adults, reflecting a more conservative gait strategy (Laudani et al. 2021). The increased time spent on both feet and maintenance of greater safety margins are plausible compensation mechanisms for reduced strength and diminished sensorimotor integration in order to reduce their risk of falling (Menz et al. 2003; Schrager et al. 2008). The linear mixed-effects model revealed an interaction effect between older adults and BW060 condition in MoS_AP_, suggesting that older adults adapted differently to this weight-bearing condition than their younger counterparts. A well-documented characteristic of aging is reduced ankle strength, which often leads older adults to shift from an ankle-dominated approach to a mixed strategy (Begue et al. 2021; Laudani et al. 2021). Rather than correcting instability at the ankle joint, they engage the hip muscles as a compensatory alternative. This shift results in larger, more proximal movements of the trunk and pelvis, rather than finer corrective adjustments at the ankle. Additionally, older adults may have a reduced capacity for regulating precise foot placement, as indicated by their more consistent mean stride length between conditions – an adaptation that could be observed in younger participants. Instead, older adults appeared to compensate by prioritizing CoM control over foot placement. While this hip-driven strategy enhances stability, it comes at the cost of increased energy expenditure and potentially slower reaction times. In the medio-lateral direction, this study found no significant group differences in step width or MoS_ML_ between young and older adults, which was in contrast to previous findings (Yamaguchi and Masani 2022). This discrepancy might be attributable to differences between treadmill and overground walking. Additionally, the bodyweight support system used in this study likely restricted medio-lateral sway, which may have masked potential age-related differences in lateral spatiotemporal gait parameters and MoS_ML_.

### Gait variability

In the BW060 condition, gait variability significantly increased for all three temporal parameters. The increased variability observed during bodyweight unloading likely reflects the greater challenge posed by reduced weight-bearing over increased loading, necessitating a more active gait control strategy (Collins and Kuo 2013). In novel or demanding conditions, the nervous system often increases variability as an adaptive mechanism, allowing for exploration and fine-tuning of movement patterns to meet new stability demands (Abram et al. 2022). Conversely, step width variability significantly decreased under bodyweight unloading conditions. Previous research indicates that both excessive and insufficient step width variability are linked to reduced gait stability in older adults, suggesting an optimal level of variability for effective motor performance (Brach et al. 2005; Hausdorff 2005; König et al. 2016; Ravi et al. 2020). Given the unfamiliarity of bodyweight unloading, participants may have prioritized more rigid control of lateral stability, potentially resulting in secondary compensation mechanisms and limiting natural variability (Kuo and Donelan 2010).

For bodyweight loading conditions, no significant differences were observed. One possible explanation is that the additional load was not sufficient to induce notable gait adaptations. In daily life, individuals frequently carry loads and are therefore considerably more accustomed to weight-bearing than to bodyweight unloading. As a result, the familiar nature of loading may have required fewer adjustments, leading to minimal unplanned changes in gait patterns.

The strategies used to adapt to bodyweight unloading conditions may differ between younger and older adults due to age-related changes in sensory, motor, and neural systems. Older adults exhibited significantly greater gait variability in stride time, double- and single-limb support time, and stride length, which are all consistent with previous findings (Hausdorff 2005; Menz et al. 2003; Schrager et al. 2008). Increased gait variability often points to compromised motor regulation, which may hinder accurate foot placement and the dynamic management of body CoM (Maki 1997). Both factors are strongly associated with an increased risk of falls in older adults (Maki 1997).

### H-reflex

Our study observed a significant decrease in H-reflex amplitude under bodyweight unloading conditions in young participants with healthy regulation of postural control. Reduced H-reflex amplitudes indicate a task-specific adaptation of the sensorimotor pathways (Theodosiadou et al. 2023), however, the precise origin of this modulation remains unclear. The unfamiliar bodyweight unloading condition might necessitate greater supra-spinal involvement to maintain stability, shifting motor control away from automatic reflex-driven adjustments towards more active regulation (Dragunas and Gordon 2016; Skiadopoulos et al. 2020). Additionally, alterations in sensory feedback (e.g. haptic, proprioceptive) under reduced loading conditions may only provide limited support to normal postural control mechanisms, hence requiring further active neural adaptations (Marchant et al. 2020). In contrast, the observed increase in H-reflex amplitude in BW140 in young adults leads to greater lower limb muscle activation and stiffness (Ferris et al. 2001; Tsuruike et al. 2012). This adaptation could help regulate reflex amplitudes under higher mechanical loads, ensuring adequate postural stability, while managing the increased demands placed on the neuromuscular system.

No significant overall differences in H-reflex amplitudes were observed between groups. However, a significant interaction effect in the BW060 condition suggests that older adults respond differently to bodyweight unloading compared to younger adults. Additionally, a trend toward reduced reflex modulation in older adults was evident, aligning with previous research showing diminished H-reflex adaptability with aging (Chalmers and Knutzen 2000; Kido et al. 2004; Scalia et al. 2024; Tsuruike et al. 2012). These findings indicate that older adults may rely less on reflex-driven adjustments and instead compensate with alternative gait strategies that may depend more on supra-spinal input (Weaver et al. 2012). However, this raises a key question: Do older adults primarily rely less on reflex control mechanisms, necessitating other control strategies? Or is reflex modulation itself diminished as a consequence of varying weight bearing conditions, thereby increasing the demand for alternative strategies to maintain stability? There are trends toward reduced H-reflex excitability in older compared to young adults. Possible explanations include the loss of afferent fibres, nerve demyelination, alterations at the Ia afferent-motoneuron synapse, or increased supra-spinal input (Theodosiadou et al. 2023). If H-reflex modulation is reduced, rapid gait adaptations to changing environments might become impossible, increasing the risk of falls. Further studies are needed to determine whether reduced reliance on reflex control mechanisms is a compensatory strategy or a direct consequence of weight perturbations requiring other gait strategies. Moreover, the reduced reflex modulation, particularly under the unfamiliarity of bodyweight unloading, is supported by the greater stride length variability in older compared to younger adults. Reduced stride length variability suggests a diminished ability to regulate step-to-step consistency in antero-posterior direction, potentially leading to less stable gait patterns and an increased reliance on supra-spinal control mechanisms to maintain balance. In contrast, step width variability did not significantly differ between groups, supporting the idea that medio-lateral balance control relies mainly on sensory integration and active neural regulation, whereas sagittal plane gait control is more dependent on feedforward mechanisms and reflex contributions (Kuo and Donelan 2010).

No significant differences were found when H-reflex amplitudes were normalized to bEMG. Changes in bEMG may have masked corresponding changes in the H-reflex, given the overall low amplitude of the signals. In literature, H-reflex normalized to bEMG is often considered a measure of afferent pathway gain within the spinal circuitry (Zehr 2002). However, given the disproportionately large changes in bEMG across different weight conditions, which serve to maintain gait stability and efficiency, these findings should be interpreted with caution. H-reflex amplitudes likely reflect task-dependent effectiveness of Ia afferents in discharging motoneurons, rather than solely representing spinal excitability (Kim et al. 2024).

### Limitations

The study setup was complex, and several limitations should be acknowledged. Measurements were conducted on a treadmill, which may not fully replicate the biomechanics of overground walking, where step width tends to be more pronounced and gait variability is typically lower (Fallahtafti et al. 2021; Hollman et al. 2016; Rosenblatt and Grabiner 2010). Participants were also required to walk at a fixed speed. However, instead of adjusting step width or walking speed, they may have used other strategies, such as foot placement or CoM regulation to adjust their gait patterns as seen in stride length and timing changes. Furthermore, using foot markers for defining the boundaries of the BoS instead of the CoP tends to overestimate the MoS and does not account for medio-lateral ankle strategies. Moreover, while the bodyweight support system imposed movement constraints, particularly in medio-lateral direction, it effectively reduced loading and is also associated with reducing effort, fear, and anxiety among older adults (Thomas et al. 2007). The constraints in the medio-lateral direction may have restricted natural hip abduction and step width adjustments, potentially explaining the lack of step width adaptations. Additionally, when comparing H-reflex amplitudes across age groups, habitual physical activity levels should be considered (Theodosiadou et al. 2023), as they can influence neuromuscular function, however, this variable was not assessed in the present study. Finally, as the H-reflex can be influenced by caffein (Jacobson and Edwards 1990), the intake of caffeinated beverages such as coffee or tea was a potential confounding factor that was not specifically controlled in the present study. While this complex experimental setup may not have allowed complete movement freedom, it did allow for the capture of extended and controlled walking protocols.

### Outlook

Achieving stable gait patterns requires seamless integration of feedforward, feedback and reflex control mechanisms to produce coordinated motor output that responds effectively to disturbances. Older adults adopted more conservative gait strategies with larger double-limb support time, reduced stride length, and increased MoS_AP_, characterized by increased safety margins and reduced adaptability of reflex modulation. Thus, prevention strategies should aim to maintain automaticity and the optimal functionality of as many control systems as possible (Clark 2015). While individual systems can compensate for impairment of one control system, the failure of multiple systems significantly diminishes an individual’s ability to recover from disturbances, hence increasing the risk of falls (Kuo 2005). Understanding these mechanisms provides a foundation for developing targeted prevention strategies, such as reactive control or balance training, to enhance stability and minimize fall risk in vulnerable populations. A comprehensive approach that trains multiple systems simultaneously may yield the best outcomes. For instance, treadmill training under varying weight-bearing conditions combined with controlled perturbations in a protective environment could effectively improve the complex interactions involved in sensorimotor integration. This multifaceted approach could enhance overall system coordination, ultimately reducing the risk of falls and promoting greater stability.

## Conclusion

This study highlights how varying weight-bearing conditions influence gait adaptations, gait variability, and reflex control mechanisms in young and older adults. The reduced antero-posterior margin of stability with bodyweight unloading and the minimal H-reflex modulation in older adults suggest age-related limitations in spinal-level adaptations. These results emphasize a shift toward different gait strategies in older adults, underscoring the need to consider neuromuscular adaptations when designing prevention programs to improve stability and reduce fall risk.

## Supplementary Information

Below is the link to the electronic supplementary material.

Supplementary file1 (PDF 296 kb)

## Data Availability

The datasets generated during and/or analyzed during the current study are not publicly available due to privacy or ethical restrictions, but are available from the corresponding author on reasonable request.

## Abbreviations

BoS: Base of support
BW060: 40% bodyweight unloading
BW080: 20% bodyweight unloading
BW100: Normal bodyweight
BW120: 20% bodyweight loading
BW140: 40% bodyweight loading
CoM: Centre of mass
EMG: Electromyography
MoS: Margin of stability
MoS_AP_: Antero-posterior margin of stability
MoS_ML_: Medio-lateral margin of stability
SD: Standard deviation
XCoM: Extrapolated centre of mass
