# Supplementary material for: Probing gait adaptations: The impact of aging on dynamic stability and reflex control mechanisms under varied weight-bearing conditions
Source: Eur J Appl Physiol. 2025 Jul 1;125(12):3753–67. doi: 10.1007/s00421-025-05884-1 (PMC12678455; doi:10.1007/s00421-025-05884-1)
Supplement: Supplementary file 1 — Supplementary file1 (PDF 296 kb) [file 421_2025_5884_MOESM1_ESM.pdf]

European Journal of Applied Physiology

Supplementary Material

PROBING GAIT ADAPTATIONS: THE IMPACT OF AGING ON DYNAMIC STABILITY AND REFLEX CONTROL UNDER VARIED WEIGHT-BEARING CONDITIONS

Michelle Gwerder[1], Ursina Camenzind[1], Samira Wild[1], Yong K Kim[1], William R Taylor[1], Navrag B Singh[1,2]

[1]Laboratory for Movement Biomechanics, Department of Health Sciences and Technology, ETH Zürich, Zürich, ZH, Switzerland
[2]Singapore-ETH Centre, Future Health Technologies Program, CREATE Campus, Singapore, Singapore

michelle.gwerder@hest.ethz.ch

1. Marker Set

Name	Segment	Location
RTO1	Foot - right	Caput metatarsale I
RTO3	Foot - right	Metatarsale III
RTO5	Foot - right	Caput metatarsale VI
RFOX	Foot - right	Lateral, not specified (Extramarker)
RHEE	Foot - right	Calcaneus
RMMA	Shank - right	Medial malleolus
RLMA	Shank - right	Lateral malleolus
RTMT	Shank - right	unspecified Tibia, ventral
RTLF	Shank - right	unspecified Fibula, lateral
RTTT	Shank - right	Tuberositas tibea
RTIB	Shank - right	Caput fibulae
RMCO	Thigh - right	Medial femur epicondylus
RLCO	Thigh - right	Lateral femur epicondylus
RTFR	Thigh - right	unspecified Femur, front distal
RTLL	Thigh - right	unspecified Femur, lateral distal
RTLH	Thigh - right	unspecified Femur, lateral proximal
LTO1	Foot - left	Caput metatarsale I
LTO3	Foot - left	Metatarsale III
LTO5	Foot - left	Caput metatarsale VI
LHEE	Foot - left	Calcaneus
LMMA	Shank - left	Medial malleolus
LLMA	Shank - left	Lateral malleolus
LTMT	Shank - left	unspecified Tibia, ventral
LTLF	Shank - left	unspecified Fibula, lateral
LTTT	Shank - left	Tuberositas tibea
LTIB	Shank - left	Caput fibulae
LMCO	Thigh - left	Medial femur epicondylus
LLCO	Thigh - left	Lateral femur epicondylus
LTFR	Thigh - left	unspecified Femur, front distal
LTLL	Thigh - left	unspecified Femur, lateral distal
LTLH	Thigh - left	unspecified Femur, lateral proximal
RASI	Pelvis	Spina illiaca ant. sup. – right
RTMS	Pelvis	Iliaca – right
RPSI	Pelvis	Spina illiaca post. Sup – right

SACR	Pelvis	Sacrum
LPSI	Pelvis	Spina illiaca ant. sup. – left
LTMS	Pelvis	Iliaca - left
LASI	Pelvis	Spina illiaca post. Sup - left
RSHO	Shoulder	Acromion - right
MSTC	Shoulder	Manubrium sterni cranial
CVC7	Shoulder	C7
LSHO	Shoulder	Acromion - left
RWRA	Forearm - right	Proc. styloideus radii
RWUL	Forearm - right	Proc. styloideus ulnae
RFRA	Forearm - right	unspecified forearm, radial
RFUL	Forearm - right	unspecified forearm, ulnae
RMEC	Humerus - right	Medial epicondylus
RLEC	Humerus - right	Lateral epicondylus
RHVT	Humerus - right	unspecified upperarm, ventral
RHLT	Humerus - right	unspecified upperarm, lateral
LWRA	Forearm - left	Proc. styloideus radii
LWUL	Forearm - left	Proc. styloideus ulnae
LFRA	Forearm - left	unspecified forearm, radial
LFUL	Forearm - left	unspecified forearm, ulnae
LMEC	Humerus - left	Medial epicondylus
LLEC	Humerus - left	Lateral epicondylus
LHVT	Humerus – left	unspecified upperarm, ventral
LHLT	Humerus - left	unspecified upperarm, lateral
RFHD	Head	Headband
RBHD	Head	Headband
LFHD	Head	Headband
LBHD	Head	Headband

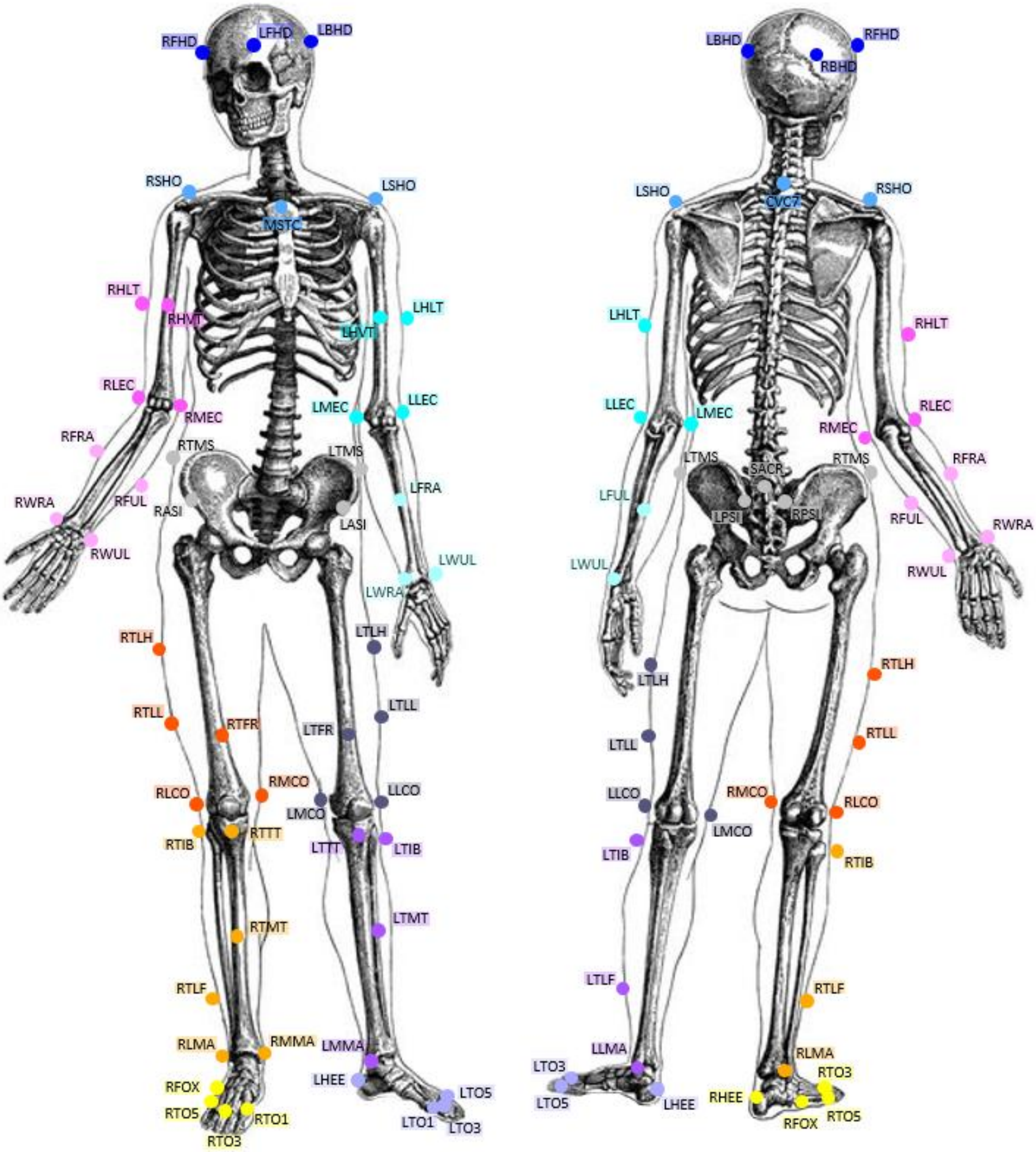
RFHD
LFHD
LBHD
RSHO
LSHO
MSTC
RHLT
RHVT
LHLT
LHVT
RLEC
RMEC
LMEC
LLEC
RFRA
RTMS
LTMS
LFRA
RWRA
RFUL
RASI
LASI
RWUL
LWUL
LWRA
RTLH
LTLH
LTLL
RTLL
RTFR
LTFR
RMCO
RLCO
LLCO
LMCO
RTIB
RTTT
LTTT
LTIB
RTMT
LTMT
RTLF
RLMA
RMMA
LMMA
LHEE
RFOX
LTO5
RTO5
RTO1
LTO1
LTO3
RTO3
LBHD
RFHD
RBHD
LSHO
CVC7
RSHO
LHLT
RHLT
LLEC
LMEC
RLEC
RMEC
LTMS
SACR
RTMS
RFRA
LFUL
LPSI
RPSI
RFUL
RWRA
LWUL
RWUL
LTLH
RTLH
RTLL
LTLL
LLCO
RMCO
RLCO
LMCO
LTIB
RTIB
LTLF
RTLF
LLMA
RLMA
LTO3
RTO3
LTO5
LHEE
RHEE
RFOX
RTO5

2. Statistical Analysis

S1: Linear mixed effects model results for mean gait parameters (green positive and red negative correlation (* p<0.05, ** p<0.01, *** p<0.001)). Young group and BW100 were set as intercept. MoS_{AP}: antero-posterior margin of stability; MoS_{ML}: mediolateral margin of stability.

Term	Stride Time	Single Support	Double Support	Stride Length	Step Width	MoS_{AP}	MoS_{ML}
(Intercept)	1.1283***	0.4143***	0.3017***	118.8485***	9.3506***	156.1576***	97.8199***
Group	0.0785	0.0146	0.0519*	-17.0092***	0.2886	34.5585**	-0.3063
BW060	-0.0053	0.0680***	-0.1383***	-2.9996**	-0.5606	-91.2928***	-5.6674
BW080	-0.0027	0.0323***	-0.0683***	-1.5079	-0.9890*	-47.2043***	-6.9294*
BW120	-0.0004	-0.0069	0.0121	0.2034	-0.3116	4.5763	1.1257
BW140	-0.0085	-0.0166**	0.0248**	-0.2929	-0.0277	3.7621	1.9357
Group:BW060	0.0150	-0.0080	0.0223	1.7606	0.1448	26.4259***	0.9742
Group:BW080	0.0050	-0.0004	0.0048	0.0223	-0.4919	6.9781	-0.2303
Group:BW120	-0.0049	-0.0022	-0.0006	-0.3879	-0.2580	1.6140	-1.3142
Group:BW140	-0.0041	-0.0019	-0.0013	-0.0916	-0.3627	1.6252	-0.4355
R^2	0.95	0.92	0.92	0.97	0.86	0.93	0.84

S2: Linear effects model results for variability gait parameters (green positive and red negative correlation (* p<0.05, ** p<0.01, *** p<0.001)). Young group and BW100 were set as intercept. SD: standard deviation; MoS_{AP}: antero-posterior margin of stability; MoS_{ML}: mediolateral margin of stability.

Term	Stride Time SD	Single Support SD	Double Support SD	Stride Length SD	Step Width SD	MoS_{AP} SD	MoS_{ML} SD
(Intercept)	0.0159***	0.0088***	0.0112***	2.8185***	1.9311***	13.2810***	8.8408***
Group	0.0130**	0.0060*	0.0073*	0.7770*	0.2384	3.3643	1.9990
BW060	0.0051*	0.0070***	0.0060***	0.2370	-0.6116**	1.0714	3.0492***
BW080	0.0006	0.0016	0.0010	-0.2059	-0.4511*	-1.0422	0.8389
BW120	0.0009	-0.0002	0.0000	0.0656	0.1594	0.8918	0.4871
BW140	0.0003	-0.0005	0.0011	0.0807	0.2154	1.7636	1.0078
Group:BW060	-0.0040	-0.0015	-0.0024	-0.2530	0.0612	-0.1038	-1.1571
Group:BW080	-0.0036	-0.0019	-0.0015	-0.3172	-0.2114	0.8461	0.0750
Group:BW120	-0.0015	0.0005	-0.0006	-0.1363	0.2057	-0.1737	-0.2688
Group:BW140	-0.0009	0.0008	-0.0013	-0.2082	0.2726	-1.6421	-0.8242
R^2	0.82	0.75	0.83	0.72	0.59	0.60	0.74

S3: Linear mixed effects model results for reflex parameters (green positive and red negative correlation (* p<0.05, ** p<0.01, *** p<0.001)). Young group and BW100 were set as intercept. bEMG: baseline EMG. H-reflex$_{norm}$: H-reflex normalized to bEMG.

**Term**	**H-reflex**	**H-reflex$_{norm}$**	**bEMG**
**(Intercept)**	1.0844***	38.8509***	0.0464***
**Group**	-0.3430	-6.3217	-0.0175
**BW060**	-0.4660***	-3.7660	-0.0227**
**BW080**	-0.3663***	-1.6460	-0.0198**
**BW120**	0.1691	-0.6362	0.0063
**BW140**	0.2615*	-5.7130	0.0163*
**Group:BW060**	0.3664*	3.0296	0.0226*
**Group:BW080**	0.3114*	10.5985	0.0158
**Group:BW120**	-0.0906	-0.7807	-0.0003
**Group:BW140**	-0.1096	1.2380	-0.0013
**R^2**	0.74	0.55	0.75
